# Pleural Effusion and Ascites in Systemic Lupus Erythematosus: A Rare Case of Pseudo-Pseudo Meigs' Syndrome

**DOI:** 10.7759/cureus.114158

**Published:** 2026-08-08

**Authors:** Jaseem Sirajudeen, Osamah F Alrawi, Arshad Muneerudeen, Marwa A Hersi, Hana Nishan, Nishan K Purayil, Vamanjore A Naushad

**Affiliations:** 1 General Internal Medicine, Hamad General Hospital, Hamad Medical Corporation, Doha, QAT; 2 Clinical Department, College of Medicine, QU Health, Qatar University, Doha, QAT; 3 Clinical Medicine, Weill Cornell Medicine - Qatar, Doha, QAT; 4 Medical Education, Hamad Medical Corporation, Doha, QAT

**Keywords:** ascites, ca-125, pleural effusion, pseudo-pseudo meigs' syndrome (ppms), serositis, systemic lupus erythematosus (sle)

## Abstract

Pseudo-pseudo Meigs' syndrome (PPMS), also known as Tjalma syndrome, is a rare manifestation of systemic lupus erythematosus (SLE) characterized by pleural effusion, ascites, and elevated cancer antigen 125 (CA-125) levels in the absence of ovarian tumors. We report the case of a 26-year-old woman who presented with dyspnea and pleuritic chest pain. On evaluation, she was found to have a left-sided pleural effusion. Further investigations confirmed SLE. Laboratory findings and imaging studies ruled out infectious and malignant causes, leading to a diagnosis of PPMS by exclusion. Treatment with prednisolone and hydroxychloroquine (HCQ) resulted in marked clinical and serological improvement, with complete resolution of the pleural effusion. Additionally, we discuss the key differences between Meigs' syndrome and PPMS and compare this case with others reported in the literature. In patients with SLE presenting with pleural effusion, ascites, and elevated CA-125 levels, PPMS should be suspected. Diagnosis involves ruling out infectious and malignant causes through fluid analysis and imaging studies. This condition usually responds well to immunosuppressive therapy.

## Introduction

Meigs' syndrome is characterized by the combination of pleural effusion, ascites, and a benign ovarian tumor and is a recognized condition typically associated with elevated cancer antigen 125 (CA-125) levels [1]. Pseudo-pseudo Meigs' syndrome (PPMS) represents a variant occurring in systemic lupus erythematosus (SLE), in which similar clinical features occur without the presence of an ovarian tumor [2]. In PPMS, pleural effusion and ascites result from serositis, a chronic inflammatory condition seen in SLE, rather than from ovarian tumor-related mechanisms. PPMS classically resolves with management of the underlying autoimmune disease [3]. We present a rare case of PPMS in a young woman that resolved completely with prednisolone and hydroxychloroquine (HCQ).

## Case presentation

A 26-year-old South African woman employed as a cabin crew member presented with a two-month history of dry cough, exertional dyspnea, and left-sided pleuritic chest pain. She also reported a history of hair loss, recurrent mouth ulcers, and morning stiffness. Her travel history included recent visits to Japan and Indonesia.

**Figure 1:**
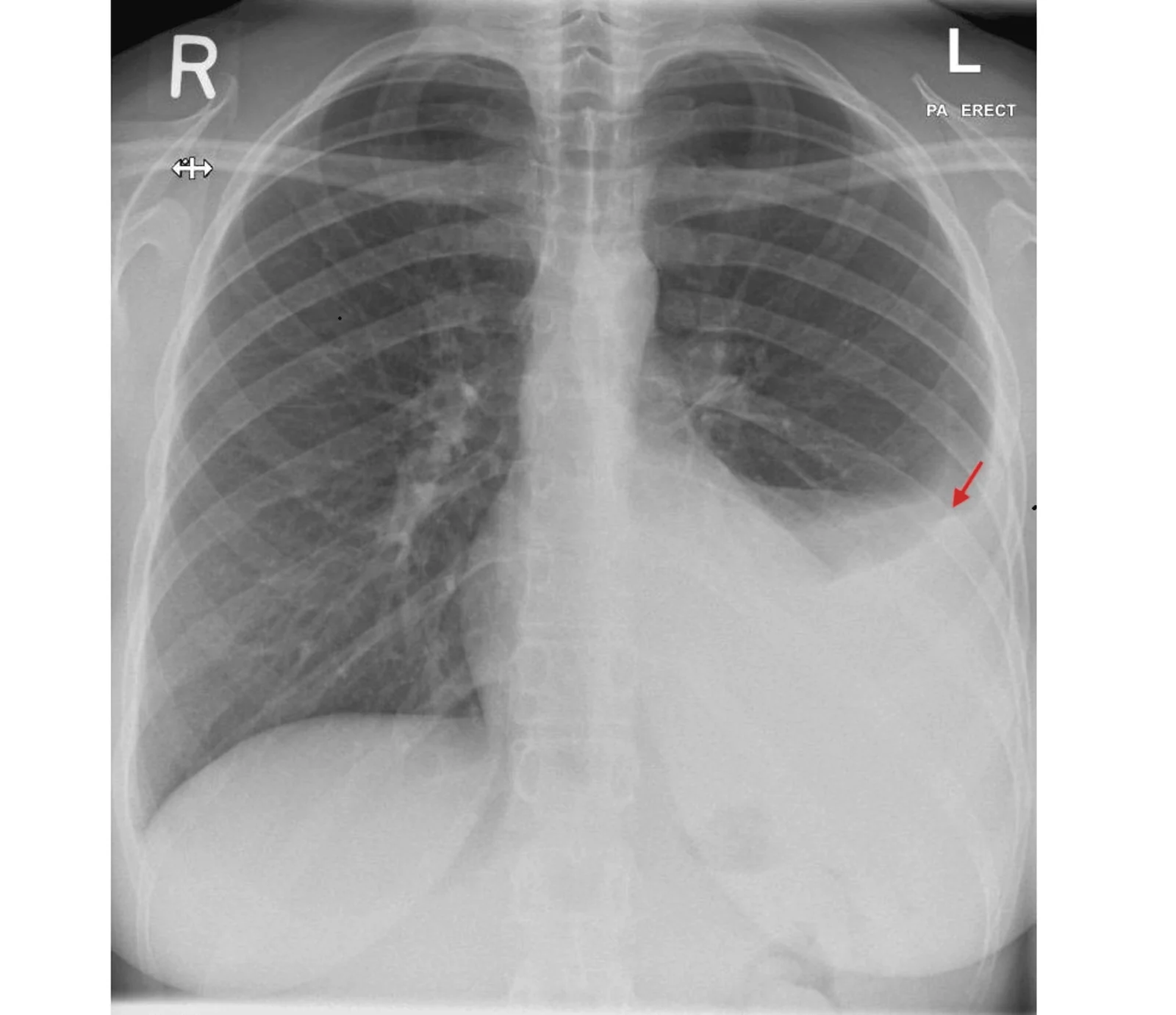
Moderate left pleural effusion with underlying lung collapse/consolidation silhouetting the left heart border (red arrow)

Physical examination revealed dullness to percussion and reduced breath sounds over the lower left lung fields. A chest X-ray demonstrated a moderate left pleural effusion (Figure 1). Initial laboratory tests showed mild normocytic normochromic anemia but were otherwise unremarkable. A diagnostic pleural tap showed an exudative, lymphocyte-predominant effusion (glucose: 5.8 mmol/L; lactate dehydrogenase (LDH): 125 U/L (vs. serum LDH of 144 U/L); protein: 47.8 g/L (vs. serum protein of 66 g/L); culture: no growth; cytology: no atypical or malignant cells). Despite extensive evaluation, including cytology, immunohistochemistry, tumor marker assessment (with the only significant finding being an elevated CA-125 level of 156 U/mL), and pleural biopsy, malignancy was excluded.

Autoimmune workup revealed a positive antinuclear antibody (ANA) test, elevated anti-double-stranded DNA (anti-dsDNA) antibody levels, and low complement levels (C3 and C4) (Table 1).

**Table 1:** Relevant blood investigation results CTD, connective tissue disease; ANA, antinuclear antibody; dsDNA, double-stranded DNA; SSA, Sjögren syndrome type A; SSB, Sjögren syndrome type B

Test	Result	Lab reference range
Antinuclear antibody CTD	Positive	
Anti-dsDNA antibody	>379.00 IU/mL	Negative <10; equivocal 10-15; positive >15
Anti-dsDNA antibody int	Positive	Negative <10; equivocal 10-15; positive >15
Anti-nuclear antibody (ANA)	Positive	N/A
ANA titer	Positive 1:1280	N/A
ANA pattern	Homogeneous	N/A
Complement component C3	0.50 gm/L	0.90-1.80
Complement component C4	0.05 gm/L	0.10-0.40
Cancer antigen 125	156.0 U/mL	0.0-35.0
Anti-La (SSB) antibody	24 U/mL	<1.0 U
Anti-La antibody int	Positive	Negative <7; equivocal 7-10; positive >10
Anti-Ro (SSA) antibody	>240.0 U/mL	<1.0 U
Anti-Ro antibody int	Positive	Negative <7; equivocal 7-10; positive >10

**Figure 2:**
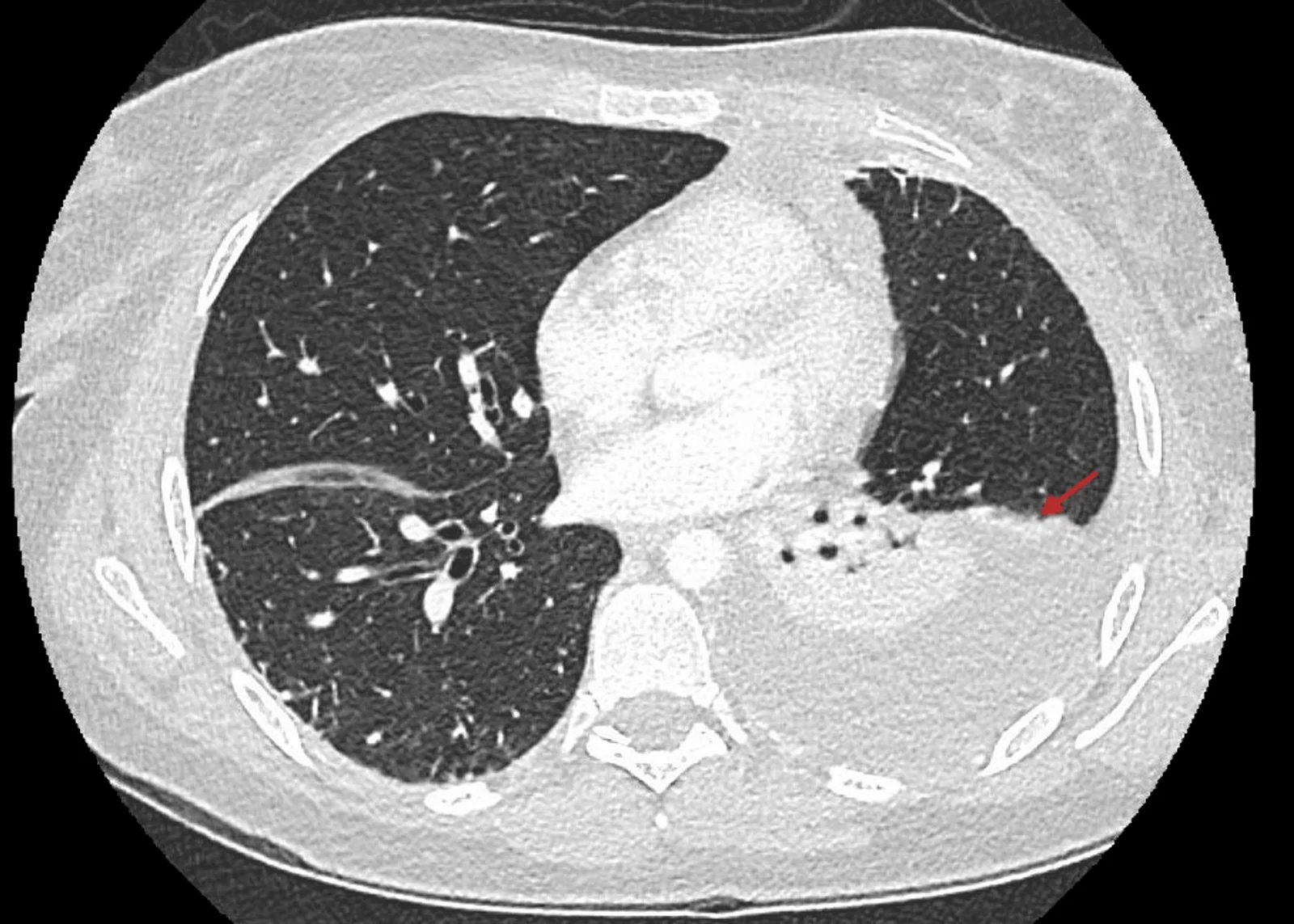
Contrast-enhanced CT of the thorax showing moderate left pleural effusion with underlying lung collapse and air bronchograms secondary to passive compressive atelectasis (red arrow) CT, computed tomography

Contrast-enhanced computed tomography (CT) of the thorax demonstrated a moderate left pleural effusion with underlying lung collapse and an air bronchogram secondary to passive compressive atelectasis (Figure 2).

**Figure 3:**
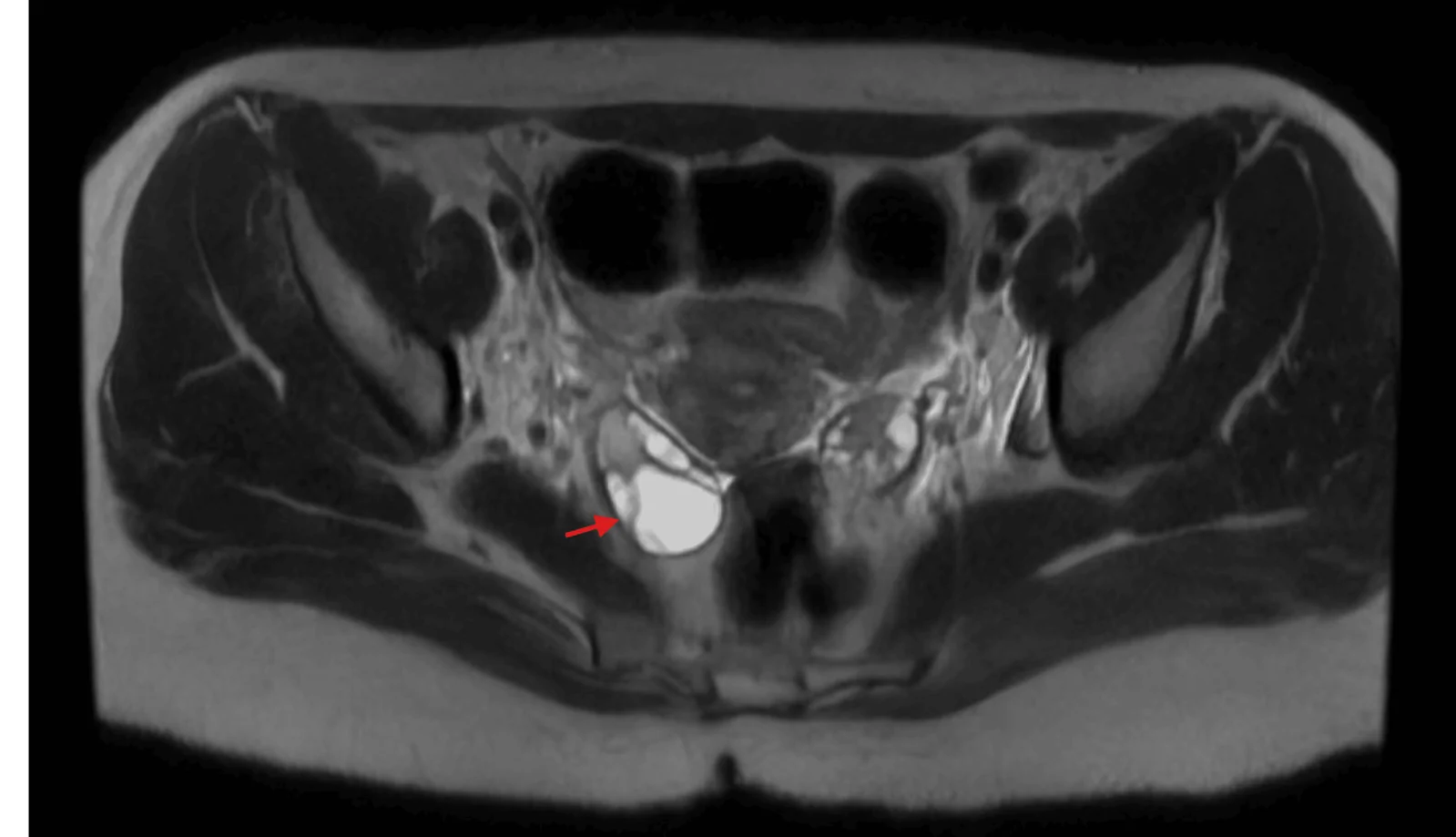
Contrast-enhanced MRI of the abdomen showing a 19×20 mm septated cyst in the right ovary, classified as O-RADS 3 (red arrow) MRI, magnetic resonance imaging; O-RADS, Ovarian-Adnexal Reporting and Data System

Furthermore, contrast-enhanced magnetic resonance imaging (MRI) of the abdomen revealed mild ascites and a 19×20 mm septated cyst in the right ovary with an Ovarian-Adnexal Reporting and Data System (O-RADS) classification of 3, signifying a low risk of malignancy (Figure 3).

She was diagnosed with SLE and serositis based on her clinical presentation and serological findings, satisfying the 2019 American College of Rheumatology/European Alliance of Associations for Rheumatology (ACR/EULAR) classification criteria with a score of 17 (Table 2).

**Table 2:** 2019 ACR/EULAR criteria score for systemic lupus erythematosus ANA, antinuclear antibody; C3, complement component 3; C4, complement component 4; dsDNA, double-stranded DNA; ACR, American College of Rheumatology; EULAR, European Alliance of Associations for Rheumatology

Satisfied criteria	Score
Positive ANA	Entry criteria
Oral ulcers	2
Pleural effusion	5
Low C3 and C4	4
Anti-dsDNA	6
Total score	17

Notably, the absence of an ovarian tumor (clinically, the patient had no symptoms or signs of an ovarian mass, and imaging showed only an incidental ovarian cyst lacking the characteristics of a fibroma or fibrothecoma), combined with the presence of pericardial effusion, ascites, and pleural effusion, suggested the possibility of PPMS.

The patient was started on prednisolone 40 mg daily for two weeks (then tapered and discontinued over four to six weeks). The initial dose of HCQ was 200 mg twice daily (later reduced to once daily). This resulted in significant clinical improvement and resolution of the pleural effusion and ascites. This response, combined with the exclusion of malignancy as a potential etiology, further supported the diagnosis of SLE-related PPMS.

At the four-month follow-up, the patient exhibited complete resolution of symptoms without recurrence while receiving HCQ alone. Imaging confirmed the absence of pleural effusion, ascites, and pericardial effusion. CA-125 levels were not re-evaluated during follow-up.

## Discussion

Meigs' syndrome is characterized by the triad of pleural effusion, ascites, and a benign ovarian fibroma (pseudo-Meigs' syndrome involves other ovarian tumors). It is often associated with elevated CA-125 levels in serum, pleural, and peritoneal fluids. As a diagnosis of exclusion, it requires the exclusion of ovarian carcinomas and other potential causes. A distinguishing feature of Meigs' syndrome is the complete resolution of both pleural effusion and ascites, along with normalization of CA-125 levels, following surgical resection of the ovarian tumor. To date, no cases of recurrence have been reported [1]. PPMS was first described by Tjalma et al. in 2005. Shortly thereafter, Schmitt et al. named this entity when a 33-year-old woman presented with a clinical picture of Meigs' syndrome in the context of SLE and enlarged ovaries [2]. Subsequent cases of PPMS have been reported, often without enlarged ovaries [4].

The pathophysiology of ascites and pleural effusion in Meigs' syndrome remains speculative. Ascites is thought to result from direct irritation of the peritoneum by hard, solid ovarian tumors, which triggers the production of peritoneal fluid. However, Samanth and Black noted that ovarian tumors associated with ascites are typically larger than 10 cm in diameter and feature a myxoid component within their stroma, which may indicate fluid secretion by the ovarian tumors as the cause of ascites in these cases [5]. The prevailing theory for pleural effusion in Meigs' syndrome is the movement of ascitic fluid into the pleural cavity through diaphragmatic lymphatics [1]. In contrast, PPMS is thought to result from chronic serositis. This difference in the underlying mechanisms necessitates different management strategies. While the primary treatment for Meigs' syndrome is surgical resection of the ovarian tumor, PPMS is managed by treating the underlying SLE with glucocorticoids and immunosuppressive agents [3].

A review of the literature on PPMS cases revealed a pattern in its diagnosis. One case described a 28-year-old woman with no prior medical history who developed symptoms following an influenza infection. Initial complaints of dry mouth, facial edema, and scleral edema were accompanied by laboratory findings consistent with Sjögren's syndrome and hypoalbuminemia. Despite treatment with albumin supplementation and diuretics, her condition deteriorated over two weeks, manifesting as generalized edema, fatigue, alopecia, and a malar rash. Subsequent investigations confirmed SLE, with imaging revealing bilateral pleural effusions and significant ascites. Laboratory results showed elevated CA-125 levels (ascitic fluid: 858 U/mL, pleural fluid: 325 U/mL, serum: 1225 U/mL). Treatment included glucocorticoids, mycophenolate mofetil (MMF), and telitacicept, leading to significant improvement after two weeks and discharge. One month later, residual ascites persisted, but the pleural effusion had resolved, complement levels had normalized, and the serum CA-125 level had decreased to 98.5 U/mL [4].

In the second case, a 37-year-old woman with a decade-long history of Sjögren's syndrome, managed with glucocorticoids and HCQ, presented with ascites, bilateral pleural effusions, reduced urine output, and a small pericardial effusion. Investigations confirmed SLE and lupus nephritis. Subsequent analysis of the peritoneal fluid revealed a CA-125 level of 359 U/mL and excluded infectious causes. The patient was treated with glucocorticoids, MMF, and telitacicept. Over time, she achieved complete resolution of symptoms, including ascites, and normalization of CA-125 levels. However, her lupus nephritis progressed to the chronic phase [4].

The first case shares similarities with our patient in that both had no significant medical history, although their presenting complaints differed. In the second case, the patient had a long history of autoimmune disease, but like our patient, she presented with a small pericardial effusion, likely due to serositis. In all these cases, PPMS resolved, and CA-125 levels normalized (not retested in our case) following treatment of the underlying SLE.

The diagnostic approach to PPMS involves confirming the presence of SLE, pleural effusion, and ascites. The next step is to exclude infectious and malignant causes through analysis of pleural fluid, peritoneal fluid, serum, or a combination of these. Elevated CA-125 levels can raise concern for ovarian neoplasms; however, if imaging excludes malignant etiologies and fluid analysis rules out infection, a diagnosis of PPMS by exclusion can be made. This diagnosis is further supported by resolution of the condition following treatment of the underlying SLE. Therefore, in patients with SLE, ascites, and pleural effusion, PPMS should be strongly suspected because it is a benign condition that can be effectively managed with appropriate treatment.

A possible explanation for CA-125 elevation is activation of mesothelial cells by inflammatory cytokines and irritation from pleural and ascitic fluid, with autoantibodies triggering these mechanisms [6].

Lupus pleuritis usually produces bilateral, small-to-moderate pleural effusions. It is uncommonly unilateral and large and is often misdiagnosed as tuberculous pleurisy [7]. The same applies to PPMS. Our patient therefore had the rare presentation of a predominantly unilateral pleural effusion, which prompted an extensive tuberculosis workup that was negative.

## Conclusions

In patients with SLE presenting with pleural effusion, ascites, and elevated CA-125 levels, PPMS should be suspected. Diagnosis involves ruling out infectious and malignant causes through fluid analysis and imaging studies. The condition can be effectively managed with glucocorticoids and immunosuppressive agents, leading to complete resolution, as demonstrated in our case. Timely identification and treatment of the underlying SLE are key to achieving favorable outcomes in PPMS.

Normalization of CA-125 is frequently reported in PPMS. However, it was not reassessed because it was deemed clinically unnecessary. A repeat CA-125 measurement and/or follow-up imaging of the ovarian cyst would have substantially strengthened the diagnostic certainty of PPMS.

## References

[1] Riker D, Goba D (2013). Ovarian mass, pleural effusion, and ascites: revisiting Meigs syndrome. J Bronchology Interv Pulmonol.

[2] Schmitt R, Weichert W, Schneider W, Luft FC, Kettritz R (2005). Pseudo-pseudo Meigs' syndrome. Lancet.

[3] Li T, Xie QB (2019). A case report of pseudo-pseudo Meigs' syndrome. Chin Med J (Engl).

[4] Li Q, Tian B (2024). Case report: three cases of systemic lupus erythematosus presenting primarily with massive ascites and significantly elevated CA-125 levels and a review of pseudo-pseudo Meigs' syndrome in literature. Front Immunol.

[5] Samanth KK, Black WC 3rd (1970). Benign ovarian stromal tumors associated with free peritoneal fluid. Am J Obstet Gynecol.

[6] Hussein AK, Aljbour OA, Aljbour JA, Alshaer HA, Alshami SK (2025). A rare case of polyserositis with elevated serum cancer antigen (CA)-125 in systemic lupus erythematosus: a case report of pseudo-pseudo Meigs syndrome. Cureus.

[7] Ren M, He Y, Zhang J, Zhang J, Zhang G (2024). Generalised blisters and respiratory distress: a case of bullous systemic lupus erythematosus. Clin Cosmet Investig Dermatol.

